# Micrococci in Relation to Wounds, Abscesses, and Septic Processes

**Published:** 1884-12

**Authors:** W. Watson Cheyne


ARTICLE VI.

### MICROCOCCI IN RELATION TO WOUNDS, ABSCESSES, AND SEPTIC PROCESSES.

Mr. W. Watson Cheyne, in his report on this subject to the Scientific Grants Committee of the British Medical Association, thus sums up the chief points of interest in his paper:

1. There are various kinds of micrococci found in wounds treated aseptically, differing markedly from each other in their effects on animals. They agree in growing best at the temperature of the body, and in causing acidity and sweatly smell in the fluids in which they grow. The experiments show that cultivations may be carried on in fluids with accuracy, provided the precautions mentioned be observed.

2. The micrococci tested in these experiments grew best in materials exposed to oxygen gas. They grow only with difficulty in the absence of oxygen. Eggs were not good pabulum.

3. Their effect on animals was not altered by growth with or without oxygen.

4. The effects of these micrococci; on rabbits and man were not similar, some of the most virulent forms for rabbits causing no deleterious effects in wounds in man.

5. The kidney is apparently an important excreting organ for organisms.

6. Organisms not capable of growing in the blood may yet cause serious effects by growing in the excretory canals. This may explain some bases of pyelitis.

Where an organism is not markedly pathogenic, it may be necessary to introduce a large quantity before morbid changes are set up.

8. Suppuration is not always due to micrococci; it may be caused by chemical irritants, such as croton oil.

9. Micrococci are always present in acute abscesses, and are probably the cause of them.

10. In some cases the micrococci are the primary cause of the inflammation and suppuration, as in pyæmic abscesses; generally, however, they begin to act after inflammation has been previously induced.

11. This inflammation may be caused by an injury, by the absorption of chemically irritating substances from wounds, by cold, etc.

12. There are several different kinds of micrococci associated with suppuration.

13. Micrococci cause suppuration by the production of a chemically irritating substance, which if applied to the tissues in a concentrated form, causes necrosis of the tissue, but if more dilute, causes inflammation and suppuration.

14. The conditions in wounds and abscesses are not the same, inasmuch as in the former there is opportunity for mechanical and chemical irritants to work.

15. There is no reason for denying the existence of "antiseptic suppuration."

16. Tension may also cause suppuration, but it is perhaps most frequently aided by the growth of micrococci. These organisms need not be of a very virulent kind. It is also probable that the products of inflammation are themselves irritating and capable of exciting or keeping up inflammation.

17. The micro-organisms of septicæmia, and erysipelas are different from one another and from those of abscesses. In erysipelas the micrococci grow in the lymphatic spaces. In pyæmia, they grow in the blood to form colonies and emboli. In septicæmia they may only grow locally, the symptoms being due to the absorption of their ptomaines; or if they grow in the blood they do not form colonies and emboli. Septicæmia may also due to other organisms besides micrococci.

18. There are no facts to support the view that it is the same micrococcus which, under different conditions, cause the various diseases. The experiments of conversion of innocent into malignant forms, and *vice versa*, are unreliable. *British Med. Journal.*

